# Comparison of Safety and Efficacy of Cefepime Administered via Intravenous Push Versus Intravenous Piggyback Infusion in Patients with Gram-Negative Bacteremia

**DOI:** 10.3390/jcm15124768

**Published:** 2026-06-19

**Authors:** Mary Fronrath, Carolyn Martz, Kristin Griebe, Michael Veve, Zachary R. Smith

**Affiliations:** 1 Department of Pharmacy, Henry Ford Hospital, Detroit, MI 48202, USA; 2 Eugene Applebaum College of Pharmacy and Health Sciences, Wayne State University, Detroit, MI 48202, USA

**Keywords:** antibacterial agents, cefepime, critical illness, drug administration routes, sepsis

## Abstract

**Introduction:** Intravenous push (IVP) beta-lactam antibiotics have been adopted during parenteral solution shortages to conserve resources. Data evaluating the safety and efficacy of cefepime administered IVP versus intravenous piggyback (IVPB) infusion in Gram-negative bacteremia remain limited. We compared clinical outcomes of cefepime administered IVP versus IVPB in hospitalized patients with Gram-negative bacteremia. **Methods:** This was a retrospective cohort study across a five-hospital health system from 1 January 2014 through 31 December 2021. Adults receiving cefepime for Gram-negative bacteremia were included. The primary outcome was a tailored desirability of outcome ranking (DOOR) composite assessed through 30 days or hospital discharge, integrating clinical cure and cefepime-associated neurologic adverse effects. Clinical cure was defined as absence of recurrent bacteremia with the index pathogen after 48 h, no antibiotic escalation, and no in-hospital mortality. **Results:** A total of 254 met the inclusion criteria (127 IVPB; 127 IVP). Baseline severity was similar between groups. The primary outcome assessed by DOOR revealed no difference between IVPB and IVP groups (*p* = 0.656). Vasopressor support during therapy was more frequent in the IVP group (22.0% vs. 10.2%, *p* = 0.011), and median hospital length of stay was longer (10 vs. 7 days, *p* = 0.020). No differences were noted in other endpoints. General ward admission (OIR [aOR] 2.563, 95% CI 1.271–5.168; *p* = 0.009) and genitourinary source of bacteremia (aOR 3.398, 95% CI 1.509–7.652; *p* = 0.003) independently predicted clinical cure. **Conclusions:** In patients with Gram-negative bacteremia, cefepime administered IVP demonstrated similar safety and efficacy to IVPB infusion.

## 1. Introduction

Beta-lactam antibiotics such as penicillins and cephalosporins are a commonly prescribed class of antibiotics to treat infections. The best predicted antibacterial pharmacodynamic effect of beta-lactams is exhibited by the time that free concentrations exceed the minimum inhibitory concentration (MIC) as a function of the dosing interval [1,2]. Medication pharmacokinetics must be considered to optimize efficacy in treatment, so beta-lactams are often administered more frequently, and intravenous (IV) formulations of beta-lactams may also be infused over longer periods of time to optimize effect against pathogens with higher MICs. Changes in administration methods may alter medication’s safety and efficacy.

In hospitalized patients, Gram-negative infections are a major cause of mortality. Gram-negative infections may lead to bacteremia, which, depending on the site of infection and source control, may be considered uncomplicated or complicated. Patients should be treated with antibiotics targeted to the causative pathogen as soon as possible. It is estimated that approximately 25–50% of bloodstream infections are caused by Gram-negative bacilli, which include Pseudomonas aeruginosa and AmpC beta-lactamase producers [3]. A common antibiotic used to treat AmpC beta-lactamase-producing pathogens is cefepime, a beta-lactam antibiotic, as it has antipseudomonal activity and withstands hydrolysis by AmpC beta-lactamases [4]. Optimizing management of bacteremia, including source control, antibiotic duration, and antibiotic dosing strategies, is key to improving patient outcomes. When shortages in medications and administration materials occur, this poses a challenge in resource utilization while maintaining medication safety and efficacy.

Shortages of medications and small-volume parenteral solutions (SVPS) require changes in practice, which include providing medications through alternative methods in order to appropriately allocate resources. The aftermath of a hurricane in September 2017 caused critical shortages of drug products and SVPS, which led to changes in practice for medication administration [5]. Considerations in determining which medications can safely be administered via alternative methods include patient safety, medication efficacy and stability, pharmacokinetics, and pharmacodynamics [6]. Prior to this SVPS shortage in 2017, cefepime was administered via intravenous piggyback (IVPB) with an infusion time of 30 min throughout the health system. In response to the shortage, practice changed to administer cefepime via intravenous push (IVP) over 3–5 min. There is a limited amount of data characterizing the safety and efficacy of this cefepime administration practice [7,8,9,10]. Cefepime has been recognized to cause neurologic adverse effects thought to be related to its ability to interact with different neurotransmitter pathways that manifest as mental status changes, confusion, seizures, or myoclonus [11].

The goal of this study was to compare the safety and efficacy of cefepime administered IVP versus IVPB in patients with Gram-negative bacteremia. The findings of this study aimed to help determine if there is a difference in clinical cure and adverse effects in patients administered cefepime IVP compared to cefepime IVPB using an ordinal desirability of outcome ranking (DOOR).

## 2. Materials and Methods

This was a multicenter, retrospective, cohort study of patients hospitalized from 1 January 2014 to 31 December 2021 and administered cefepime via IVP or IVPB as empiric or targeted therapy for Gram-negative bacteremia where cefepime was indicated. The study was conducted at a five-hospital health system in southeast Michigan and approved by the health system’s Institutional Review Board. Screening and eligibility were performed through review of the institution’s electronic medical record (EMR). Gram-negative bacteremia was identified based on blood culture results in the patient’s EMR. Patients were excluded if they were less than 18 years old, empirically administered cefepime extended-infusion over 3 h, started cefepime at an outside health system, diagnosed with end-stage renal disease (ESRD) on hemodialysis, peritoneal dialysis, or inconsistent continuous renal replacement therapy (CRRT), left the hospital against medical advice (AMA), comfort care, hospice, cognitively impaired, pregnant, or incarcerated. Patients were classified into the cefepime IVP group or the cefepime IVPB group based on the method of cefepime selected for administration for the full course of treatment. Based on the workflow of the emergency department pharmacies within the health system, compounding cefepime IVPB at the time of order verification, a single first dose of IVPB was permitted within the IVP group.

The primary outcome was a tailored DOOR endpoint that included clinical cure and neurologic adverse effects during cefepime treatment. This was assessed up to 30 days or at hospital discharge, whichever occurred first, using an ordinal clinical response [12]. Patients were characterized on a scale as seen in Table 1, where each patient was assigned one ranking for their overall outcome.

Clinical cure was defined as no recurrent bacteremia with the initial pathogen after 48 h, antibiotic escalation, or in-hospital mortality at 30 days [13]. During hospitalization, if the patient was actively receiving cefepime and was switched to a different Gram-negative therapy when a new secondary infection developed this was classified as a clinical cure because the initial pathogen did not grow in subsequent culture. Adverse effects were defined as cefepime-associated neurologic adverse effects of altered mental status, agitation, or seizures, with other causes ruled out as documented in the electronic medical record [14,15]. Frequency of antibiotic escalation from cefepime to a broader-spectrum agent after 48 h of cefepime therapy was defined as a change from cefepime to piperacillin/tazobactam, an antipseudomonal carbapenem, ceftazidime/avibactam, ceftolozane/tazobactam, meropenem/vaborbactam, aminoglycosides, cefiderocol, colistin or polymyxin B or change to extended-infusion cefepime.

Secondary outcomes included frequency of antibiotic escalation from cefepime to a broader-spectrum agent, emergence or development of cefepime resistance, time to administration of first dose of cefepime, time to defervescence, vasopressor and mechanical ventilation initiation and discontinuation, neurologic adverse effects, intensive care unit (ICU) and hospital lengths of stay, and in-hospital mortality.

Data collected included baseline demographics, comorbidities, and information related to the Pitt Bacteremia Score [16]. Infection data collected included temperature, pulse, respiratory rate, laboratory values (including basic metabolic profile and complete blood count), bacteremia source and type, culture results, and antibiotic treatment. Cefepime data collected included time to therapy, type of therapy (empiric or targeted), dosing, inpatient duration of therapy, and cefepime-related adverse effects. Cefepime dosing was guided by a standard protocol that was optimized daily by clinical pharmacists by calculating creatinine clearance using the Cockcroft–Gault equation using the most recent creatinine values. Creatinine clearance of >60 mL/min recommended a dose of 2 g every 8 h, creatinine clearance of 30–59 mL/min recommended a dose of 1 g every 8 h, creatinine clearance of 15–29 mL/min recommended a dose of 1 g every 12 h, and creatinine clearance of <15 mL/min recommended a dose of 1 g every 24 h. Maximum dosing was defined as using the highest recommended dose based on the patient’s calculated creatinine clearance. Other variables included vasopressor and mechanical ventilation initiation, antipyretic administration during time to defervescence, therapy outcomes, ICU and hospital lengths of stay, and in-hospital mortality.

Uncomplicated Gram-negative bacteremia was defined as growth of a Gram-negative organism and documentation of infection with sources of bacteremia as follows: primary bacteremia/unknown source, urinary tract, abdominal, respiratory tract, central venous catheter (CVC)/hardware with catheter/hardware removal, or skin and soft tissue. Complicated Gram-negative bacteremia was defined as sources of bacteremia as follows: endocarditis/endovascular infections, necrotizing fasciitis, osteomyelitis, abdominal abscesses and other unresolved abdominal sources requiring surgical intervention, central nervous system infections (including spinal abscess), empyema, and CVCs/hardware without catheter/hardware removal [17]. Patients with concomitant Gram-positive bacteremia or fungemia were included in the study if they received active therapy against that additional pathogen(s) within 12 h of blood culture collection.

Empiric cefepime therapy was defined as the patient receiving cefepime as the only Gram-negative therapy used within the first 48 h from time zero. Targeted cefepime therapy was defined as the patient receiving cefepime within 48 h of blood culture speciation as the first active therapy against the Gram-negative organism of interest. Time zero was defined as the time when blood cultures are collected. Gram-negative organisms of interest included Pseudomonas aeruginosa and AmpC beta-lactamase producers, including *Serratia* species (spp.), *Citrobacter* spp., *Enterobacter* spp., *Klebsiella aerogenese*, *Cronobacter* spp., *Edwardsiella* spp., *Aeromonas* spp., *Hafnia* spp., *Morganella* spp., and *Providencia* spp [17]. During the study periods, all sites utilized the same methods for blood culture handling and identification of pathogens. In 2019, rapid blood culture polymerase chain reaction was implemented.

Cefepime resistance was defined as a change in cefepime breakpoint from susceptible to intermediate or resistant, or from intermediate to resistant, based on repeat blood cultures if drawn. The minimum inhibitory concentration from the susceptibility testing was utilized to define the respective susceptibility pattern for each pathogen. Time to first dose of cefepime was defined as the time from order entry by the provider to administration documentation in the EMR. Time to defervescence was defined as the time between the first temperature >38 °C (oral, tympanic, or axillary) and temperature <38 °C for at least 24 h [10].

Descriptive statistics for numerical and nominal data were analyzed using measures of central tendency and variability. Nominal data is reported as a number and percentage and compared using a two-tailed chi-square test of two independent samples. Continuous data are reported as median and interquartile range and compared using Student’s *t*-test or Mann–Whitney U test as appropriate. With alpha set as 0.05 and beta as 0.8 and an estimated incidence of clinical failure in patients with Gram-negative bacteremia treated with IVPB beta-lactam antibiotics of 14%, a sample size of 127 patients per group would be needed to detect a 10% difference in clinical failure between groups [10]. A list of patients was pulled from the electronic medical record for patients who had an active order for cefepime and one of the included bacteria in a blood culture from the study time frame. The initial list was randomized, and patients were sequentially screened for inclusion until both groups reached 127 patients to meet the sample size of 254 patients. A stepwise backward conditional multivariable logistic regression analysis was also conducted for predictors of clinical cure. Variables with an alpha value of less than 0.2 in the univariate analysis were eligible for inclusion in the multivariable logistic regression analysis.

## 3. Results

**Figure 1:**
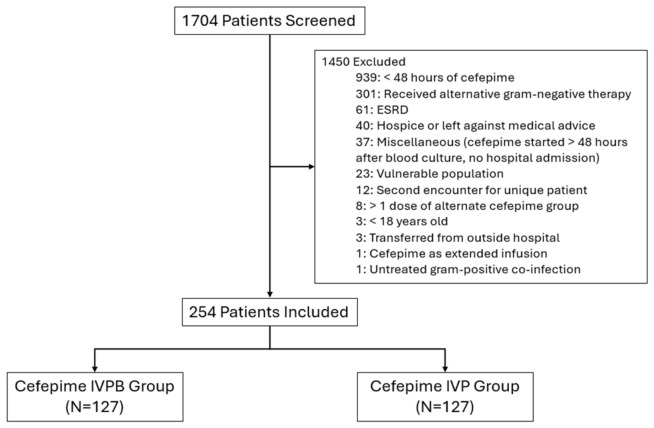
Study population screening and inclusion.

A total of 1704 patients were screened, and 1450 patients were excluded. The study population included a total of 254 patients: 127 patients in the IVPB group and 127 patients in the IVP group (Figure 1).

Baseline patient characteristics between groups were similar, with the exception of significant differences in median age and location of first cefepime dose administration. Baseline patient acuity parameters of vasopressor support and mechanical ventilation at the time of the cefepime order, Pitt Bacteremia Score, and Charlson Comorbidity Index were similar between groups. Complicated bacteremia was more common in the IVP group compared to the IVPB group (26.8% versus 15.0%; *p* = 0.021). The suspected source of infection did not differ between groups, apart from a higher rate of genitourinary infections in the IVPB group and a higher rate of lower respiratory tract infections in the IVP group. The most common pathogens between both groups were *Pseudomonas* spp., *Enterobacter* spp., *Serratia* spp., and *Citrobacter* spp. See Table 2 for additional baseline characteristics.

In both groups, cefepime dosing data were similar. The median inpatient duration of cefepime therapy was similar between the IVPB and IVP groups of 4.7 days and 5.5 days, respectively (*p* = 0.246). The administration of a single first dose of cefepime as IVPB in the IVP group occurred in 20.5% of IVP patients. Escalation of therapy from cefepime occurred in 13.4% of the IVPB group and 14.2% of the IVP group (*p* = 0.856). The most common type of escalation was antibiotic escalation in both groups. See Table 3 for an additional breakdown of cefepime data characteristics.

**Figure 2:**
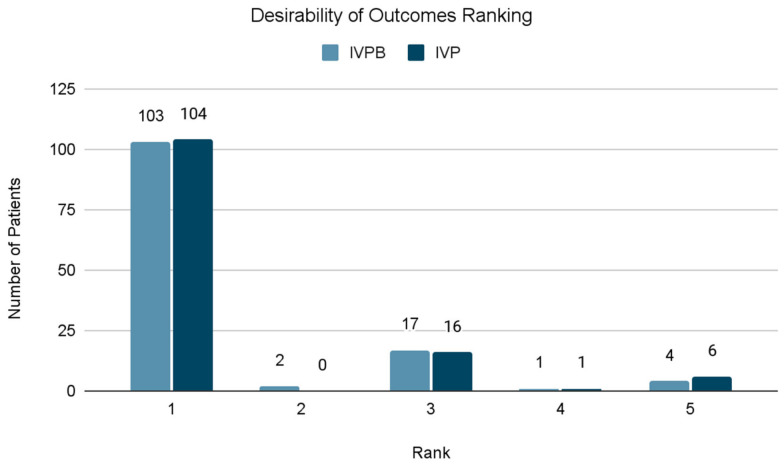
DOOR primary endpoint.

The overall DOOR endpoint distribution was similar between groups, as seen in Figure 2 (*p* = 0.656). The most common rank in both groups was rank 1 of clinical cure without adverse effects, followed by rank 3 of survival without clinical cure or adverse effects. The most common reason for clinical failure was antibiotic escalation. No differences were noted in the rates of clinical cure between the IVPB and IVP groups receiving cefepime as empiric therapy (80% (88/110) versus 80.6% (87/108), *p* = 0.918). No differences were noted in patients with *Pseudomonas aeruginosa* bacteremia between the IVPB and IVP groups with regard to clinical cure (80% (61/76) versus 77% (41/53), *p* = 0.960) and mortality (2.6% (2/76) versus 5.6% (3/53), *p* = 0.39).

When patients did not have escalation of cefepime therapy, therapy outcomes included IV-to-oral therapy change, continuing cefepime outpatient, changing to a different IV therapy that was not considered an escalation, or completing cefepime inpatient (Table 4). The only cefepime-related adverse effect that was documented was altered mental status, and this occurred in 2.4% of the IVPG group and 0.8% of the IVP group (*p* = 0.313). Vasopressor support while on cefepime therapy was more common in the IVP group compared to the IVPB group (22.0% versus 10.2%; *p* = 0.011). Median hospital length of stay was also longer in the IVP group compared to the IVPB group (7 days versus 10 days; *p* = 0.020). Other secondary endpoints were similar between groups, as shown in Table 4.

Variables assessed for association of clinical cure in the univariate analysis—defined as a DOOR of one or two—included Pseudomonas spp. as the blood pathogen, uncomplicated bacteremia, Pitt Bacteremia Score less than one, age less than 68 years, cefepime MIC < 8 ug/mL, no ICU admission, genitourinary infection as the suspected source, cefepime administered IVPB, male gender, and Charlson Comorbidity Index less than five. When these variables were included in the multivariable logistic regression model, only Pseudomonas spp. as the blood pathogen, cefepime MIC < 8 ug/mL, no ICU admission, and genitourinary infection as the suspected source were included. No ICU admission and genitourinary infection as the suspected source were independent predictors of clinical cure (Table 5).

## 4. Discussion

While treating patients with Gram-negative bacteremia with cefepime, there was no significant difference seen in instances of clinical cure or adverse effects in IVP compared to IVPB. The IVP group had more complicated bacteremia, which could contribute to the higher incidence of vasopressor use and longer median hospital length of stay. When clinical cure was not met in either group, the most common reason for this was antibiotic escalation. Multivariable logistic regression analysis showed that no ICU admission and genitourinary as the suspected source were independent predictors of clinical cure.

A previous study found no difference between groups in their primary endpoint of escalation of therapy; however, they did not have the power to assess clinical cure [10]. In order to assess the safety and efficacy of cefepime, a desirability of outcome ranking was used as the primary endpoint. Using DOOR is a novel clinical endpoint in infectious disease studies to assess strategies for optimal antibiotic use [12]. Patients are ranked by looking at both efficacy and safety outcomes, where the overall outcome is a mutually exclusive scale of hierarchical levels based on desirability in combinations of risks and benefits. A second study in critically ill patients demonstrated numerically lower rates of clinical cure in those receiving IVP cefepime when compared to IVPB [8]. The receipt of cefepime via IVP was an independent predictor of treatment failure. Taking the previous literature and our findings into consideration, further research in critically ill patients is indicated to assess if IVP cefepime is comparable to clinical outcomes to IVPB [9].

A limitation of this study includes the differences in antimicrobial resistance patterns and technology over time. As this study included patients over a timespan of eight years, changes inevitably occurred between antibiograms, MIC breakpoints for cefepime susceptibility change when assessed over time by the Clinical and Laboratory Standards Institute based on emerging evidence, and blood culture technology [18,19]. Advances within the infectious disease literature also occurred, which changed recommendations on repeating Gram-negative blood culture and the definitions for pathogens at higher risk for production of AmpC beta-lactamases [20]. This could have skewed results in how patients were treated over time based on the pathogen that grew. Based on the lower Charlson Comorbidity Indices and Pitt Bacteremia Scores amongst the patient population, these results may decrease external validity to more severely ill patients. A total of 20.5% of patients included in the cefepime IVP arm received a single dose of cefepime via IVPB. While this may have impacted the outcomes due to the potential for improved time over MIC attainment, it was felt to be a requirement given the practice in the emergency rooms to reach the appropriate sample size for the study. Rapid diagnostic blood culture systems were implemented in the health system in 2019, and the IVPB group would have been more likely to have had cultures obtained using older methods. Given that the rates of empiric therapy and pathogen susceptibility were similar between groups, it was felt that this likely would not influence the findings. Due to the timeframe of the study and the susceptibility testing employed at the study institution, the Enterobacterales isolates were classified using the historical method of susceptible (MIC < 8), intermediate (MIC: 8), and resistant (MIC > 8). Two isolates had an MIC of four, and due to the small number, this may decrease the likelihood of the impact of the contemporary classification of S-DD that requires optimized cefepime dosing. Dosing intensity of cefepime was captured on the first day of therapy, and the dose per day was not captured for the full course of therapy. Due to the retrospective nature of the present study, it may not have captured the true prevalence of cefepime-related adverse effects. A power calculation was performed based on the available literature in this area, but it is likely the study is underpowered for clinical outcomes and future research is warranted. Although patients were selected for screening from a randomized list, unmeasured differences between included and non-included patients may have limited the representativeness of the study population. The study findings are not applicable to excluded populations, notably pediatric patients and those with ESRD. Finally, by only including emergency department and inpatient administrations of cefepime, there was no follow-up on the safety and efficacy of patients who were discharged outpatient on extended courses of cefepime therapy.

## 5. Conclusions

These results demonstrate that administering cefepime IVP failed to show differences in efficacy and safety when compared to administration via IVPB. Further prospective studies should be done within a similar timeframe and in a more severely ill patient population to evaluate differences in clinical cure and adverse effects of cefepime administered IVPB versus IVP.

## Figures and Tables

**Table 1:** Clinical outcome hierarchical levels.

Rank	Outcome
1 (most desirable)	Clinical cure without adverse effects
2	Clinical cure with adverse effects
3	Survival with no clinical cure or adverse effects
4	Survival with no clinical cure but with adverse effects
5 (least desirable)	Death (any cause)

**Table 2:** Baseline characteristics.

Patient Characteristic at Cefepime Order Entry	IVPB Group (*n* = 127)	IVP Group (*n* = 127)	*p*-Value
Age, years; median (IQR)	71 (60.5–79.0)	66 (51.0–73.0)	0.004
Male; *n* (%)	89 (70.1)	74 (58.3)	0.050
Location; *n* (%)			0.030
Emergency Department	68 (53.5)	47 (37.0)	-
General Practice Unit	41 (32.3)	55 (43.3)	-
Intensive Care Unit	18 (14.2)	25 (19.7)	-
Vasopressor Support Prior to Cefepime; *n* (%)	5 (3.9)	10 (7.9)	0.718
Mechanical Ventilation Prior to Cefepime; *n* (%)	3 (2.4)	12 (9.4)	0.398
Pitt Bacteremia Score; median (IQR)	2 (0.0–3.0)	1 (0.0–3.0)	0.605
Charlson Comorbidity Index; median (IQR)	5 (4.0–7.0)	5 (3.0–7.5)	0.235
Creatinine Clearance, mL/min; median (IQR)	53.7 (36–77.2)	55.5 (32.4–101.2)	0.16
Comorbidity; *n* (%)			
Chronic Kidney Disease	50 (39.4)	48 (37.8)	0.114
Congestive Heart Failure	33 (26.0)	25 (19.7)	0.232
Stroke/Transient Ischemic Attack	24 (18.9)	14 (11.0)	0.079
Chronic Obstructive Pulmonary Disease	18 (14.2)	19 (15.0)	0.859
Diabetes Mellitus (Uncomplicated)	22 (17.3)	35 (27.6)	0.051
Diabetes Mellitus (End-Organ Damage)	14 (11.0)	12 (9.4)	0.679
Tumor (Metastatic)	14 (11.0)	17 (13.4)	0.565
Tumor (Local)	11 (8.7)	11 (8.7)	1.000
Complicated Bacteremia; *n* (%)	19 (15.0)	34 (26.8)	0.021
Bacteremia Source; *n* (%)			
Genitourinary	63 (49.6)	39 (37.0)	0.002
Central Venous Catheter	15 (11.8)	18 (14.2)	0.081
Intraabdominal	14 (11.0)	25 (19.7)	0.056
Skin and Soft Tissue	14 (11.0)	7 (5.5)	0.111
Blood/Unknown	11 (8.7)	21 (16.5)	0.059
Lower Respiratory Tract	7 (5.5)	18 (14.2)	0.021
Bone/Joint	5 (3.9)	4 (3.1)	0.734
Other	4 (3.1)	9 (7.1)	0.155
Upper Respiratory Tract	1 (0.8)	0 (0.0)	0.316
Central Nervous System	0 (0.0)	3 (2.4)	0.576
Pathogen Type; *n* (%)			
*Pseudomonas* spp.	76 (59.8)	53 (41.7)	0.004
*Enterobacter* spp.	24 (18.9)	34 (26.8)	0.135
*Serratia* spp.	10 (7.9)	22 (17.3)	0.023
*Citrobacter* spp.	10 (7.9)	15 (11.8)	0.292
*Providencia* spp.	4 (3.1)	4 (3.1)	1.000
*Morganella* spp.	3 (2.4)	1 (0.8)	0.313
*Aeromonas* spp.	1 (0.8)	0 (0.0)	0.316
*Hafnia* spp.	1 (0.8)	0 (0.0)	0.316
Pathogen MIC50	1	1	n/a
Pathogen MIC90	4	4	n/a

IQR: interquartile range; spp: species; MIC: minimum inhibitory concentration; n/a: not applicable.

**Table 3:** Cefepime data.

Cefepime Characteristic	IVPB Group (*n* = 127)	IVP Group (*n* = 127)	*p*-Value
Inpatient Duration of Therapy, days; median (IQR)	4.7 (3.0–6.5)	5.5 (3.5–7.7)	0.246
First Dose Administered IVPB; *n* (%)	127 (100)	26 (20.5)	n/a
Cefepime as Empiric Therapy; *n* (%)	110 (86.6)	108 (85.0)	0.408
Cefepime MIC < 8 ug/mL; *n* (%)	113 (89.0)	120 (94.5)	0.548
Dosing Based on Renal Function *; *n* (%)			0.408
Maximum Dosing	112 (88.2)	116 (91.3)	-
Standard Dosing	15 (11.8)	11 (8.7)	-
Escalation of Therapy; *n* (%)	17 (13.4)	18 (14.2)	0.856
Type of Escalation; *n* (%)			0.348
Antibiotic Escalation	16 (12.6)	13 (10.2)	-
Extended-Infusion Cefepime	1 (0.8)	4 (3.1)	-

* Maximum versus standard dosing based on internal health system dosing policy; n/a: not applicable.

**Table 4:** Secondary endpoints.

Secondary Endpoint	IVPB Group (*n* = 127)	IVP Group (*n* = 127)	*p*-Value
Therapy Outcome When No Escalation; *n* (%)			0.581
IV-to-Oral Therapy Change	47 (37.0)	50 (39.4)	-
Continued Cefepime Outpatient	28 (22.0)	20 (15.7)	-
IV Therapy Change, Non-Escalation	18 (14.2)	18 (14.2)	-
Completed Cefepime Outpatient	15 (11.8)	22 (17.3)	-
Cefepime-Related Adverse Effects; *n* (%)	3 (2.4)	1 (0.8)	0.313
Blood Cultures Positive >48 h; *n* (%)	3 (2.4)	2 (1.6)	0.651
Vasopressor Support While on Cefepime; *n* (%)	13 (10.2)	28 (22.0)	0.011
Mechanical Ventilation While on Cefepime; *n* (%)	8 (6.3)	18 (14.2)	0.068
Time to Defervescence, hours; median (IQR)	26.2 (24.0–38.6)	30.5 (24.0–44.3)	0.350
ICU Admission; *n* (%)	50 (39.4)	58 (45.7)	0.310
ICU Length of Stay, days; median (IQR)	4 (2.0–7.0)	5 (2.0–13.8)	0.192
Hospital Length of Stay, days; median (IQR)	7 (5.0–12.5)	10 (6.0–16.0)	0.020
In-Hospital Mortality; *n* (%)	4 (3.1)	6 (4.7)	0.519

**Table 5:** Multivariable logistic regression analysis for predicting clinical cure.

Variable	Univariate Analysis Odds Ratio (95% CI)	*p*-Value	Multivariable Analysis Odds Ratio (95% CI)	*p*-Value
*Pseudomonas* spp. as Pathogen	0.636 (0.330–1.224)	0.175	0.477 (0.226–1.006)	0.052
Uncomplicated Bacteremia	1.716 (0.826–3.565)	0.147	-	-
Pitt Bacteremia Score < 1	1.763 (0.825–3.766)	0.143	-	-
Age < 68 Years	0.556 (0.288–1.070)	0.079	-	-
Cefepime MIC < 8 ug/mL	2.985 (1.025–8.692)	0.045	3.035 (0.925–9.955)	0.067
No ICU Admission	2.380 (1.227–4.578)	0.010	2.563 (1.271–5.168)	0.009
Genitourinary Infection as Suspected Source	2.752 (1.295–5.850)	0.008	3.398 (1.509–7.652)	0.003
Cefepime Administered IVPB (versus IVP)	1.056 (0.544–2.010)	0.869	-	-
Male	1.108 (0.569–2.156)	0.764	-	-
Charlson Comorbidity Index < 5	0.768 (0.402–1.468)	0.424	-	-

## Data Availability

The datasets generated and analyzed during the current study are not publicly available due to institutional restrictions but may be obtained from the corresponding author on reasonable request.

## Abbreviations

The following abbreviations are used in this manuscript:

AMA	Against Medical Advice
AmpC	AmpC β-lactamase
CI	Confidence Interval
CRRT	Continuous Renal Replacement Therapy
CVC	Central Venous Catheter
DOOR	Desirability of Outcome Ranking
EMR	Electronic Medical Record
ESRD	End-Stage Renal Disease
ICU	Intensive Care Unit
IQR	Interquartile Range
IV	Intravenous
IVP	Intravenous Push
IVPB	Intravenous Piggyback
MIC	Minimum Inhibitory Concentration
MIC50	Minimum Inhibitory Concentration inhibiting 50% of isolates
MIC90	Minimum Inhibitory Concentration inhibiting 90% of isolates
S-DD	Susceptible-Dose Dependent
spp.	Species (plural)
SVPS	Small-Volume Parenteral Solutions
